# Immunopathology and dexamethasone therapy in a new model for malaria-associated acute respiratory distress syndrome

**DOI:** 10.1186/1475-2875-9-S2-I13

**Published:** 2010-10-20

**Authors:** Philippe E Van den Steen, Nathalie Geurts, Katrien Deroost, Ilse Van Aelst, Sebastien Verhenne, Hubertine Heremans, Jo Van Damme, Ghislain Opdenakker

**Affiliations:** 1 Laboratory of Immunobiology, Rega Institute, University of Leuven, Belgium; 2 Laboratory of Molecular Immunoloby, Rega Institute, University of Leuven, Belgium

## 

**Figure 1:**
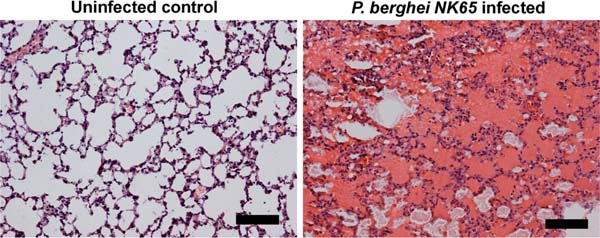
Histopathology of *P. berghei NK65-induced* MA-ARDS. Frozen sections of lungs of mice infected for 10 days with *P. berghei NK65* or control mice were stained with H&E.The black bar corresponds with 100 μm.

Malaria infection is often complicated by malaria-associated acute respiratory distress syndrome (MA-ARDS), characterized by pulmonary edema and hemorrhages. No efficient treatments are available for MA-ARDS and its pathogenesis remains poorly understood. To develop a new animal model for MA-ARDS, mice were infected with *Plasmodium berghei NK65,* and the development of MA-ARDS was characterized by increased lung weight, edema, leukocyte infiltration and hemorrhages (Figure 1). The pulmonary expression of several cytokines and chemokines was increased to a higher level than in mice infected with *P. chabaudi AS,* which does not cause MA-ARDS. By depletion experiments, CD8^+^ T lymphocytes were shown to be pathogenic. High doses of dexamethasone blocked MA-ARDS, even when administered after appearance of the complication, and reduced pulmonary leukocyte accumulation.

We developed a novel model of MA-ARDS with many similarities to human MA-ARDS and without cerebral complications. This contrasts with the more classical model with *P. berghei ANKA,* characterized by fulminant cerebral malaria. Hence, infection with *P. berghei NK65* generates a broader time window to study the pathogenesis and to evaluate candidate treatments. The finding that high doses of dexamethasone cured MA-ARDS suggests that it might be more effective against MA-ARDS than it was in the clinical trials for cerebral malaria.
